# Voice-Activated Virtual Home Assistant Use and Social Isolation and Loneliness Among Older Adults: Mini Review

**DOI:** 10.3389/fpubh.2021.742012

**Published:** 2021-10-11

**Authors:** Cynthia F. Corbett, Pamela J. Wright, Kate Jones, Michael Parmer

**Affiliations:** ^1^College of Nursing, University of South Carolina, Columbia, SC, United States; ^2^Advancing Chronic Care Outcomes through Research and Innovation Center, College of Nursing, University of South Carolina, Columbia, SC, United States; ^3^Prisma Health Senior Care Program for All-Inclusive Care for the Elderly, Greenville, SC, United States

**Keywords:** virtual home assistant, conversational assistant, voice-activated speaker, social connectedness, social isolation, loneliness, older adults, geriatric (aging)

## Abstract

A lack of social connectedness is common among older adults due to living alone, loss of loved ones, reduced mobility, and, more recently, social distancing created by the global Covid-19 pandemic. Older adults are vulnerable to social isolation and loneliness, which pose significant health risks comparable to those of smoking, obesity, physical inactivity, and high blood pressure. A lack of social connectedness is also correlated with higher mortality rates even when controlling for other factors such as age and comorbid conditions. The purpose of this mini review was to explore the emerging concepts of older adults' use of commercially available artificial intelligent virtual home assistants (VHAs; e.g., Amazon Echo, Google Nest), and its relationship to social isolation and loneliness. A secondary purpose was to identify potential areas for further research. Results suggest that VHAs are perceived by many older adult users as “companions” and improve social connectedness and reduce loneliness. Available studies are exploratory and descriptive and have limited generalizability due to small sample sizes, however, similar results were reported across several studies conducted in differing countries. Privacy concerns and other ethical issues and costs associated with VHA use were identified as potential risks to older adults' VHA adoption and use. Older adults who were using VHAs expressed the need and desire for more structured training on device use. Future research with stronger methods, including prospective, longitudinal, and randomized study designs are needed. Public education, industry standards, and regulatory oversight is required to mitigate potential risks associated with VHA use.

## Introduction

Social isolation and loneliness are serious public health concerns that affect a significant portion of the older adult population. The National Health and Aging Trends Study indicated that 24% of all community-dwelling adults aged 65 and older in the United States (US) were socially isolated (1). The National Health and Retirement Study revealed that 43% of US adults aged 60 and older report loneliness (2). Authors of a study in Germany, reported 30% of the participants expressed feeling lonely 1–3 times per month (3). Older adults are often at risk for social isolation and loneliness due to factors such as living alone, loss of loved ones, reduced mobility, vision, and hearing deficits, and, recently, the necessity for social distancing due to the Covid-19 pandemic (4). Social disconnectedness is correlated with higher mortality rates even when controlling for other factors such as age and comorbid conditions. Meta-analysis findings revealed social isolation and loneliness elevate mortality risk by 26–32%, similar in magnitude to that of established risk factors, such as smoking, obesity, physical inactivity, and high blood pressure (5). Social isolation or loneliness in older adults has also been associated with a 50% increased risk of dementia (6) and a 30% increased risk of coronary artery disease (7).

Social connection describes the structural, functional, and quality aspects of human relationships and interactions (8). Social isolation is the objective lack or limited extent of social connection with others. Loneliness is the subjective feeling of being lonely. Socially isolated people may not feel lonely, and, contrarily, persons with many social connections may express loneliness (9). Typically, social support functions to provide emotional, tangible, informational, and/or companionship assistance to improve social connection (8). Unfortunately, traditional social supports may not always be available. Although many social support interventions have been implemented in community organizations, participation by older adults may be limited by access, cost, mobility, and/or interest (8). The emergence of artificial intelligence (AI) may offer unprecedented opportunities to relieve social isolation and loneliness among older adults to improve health outcomes.

Artificial intelligence describes algorithms that emulate human cognitive and behavioral processes and are installed into software programs of various platforms connected to the internet (10). Conversational agents are one such platform, whereby a device automatically processes and responds to human voice and language. Through natural language processing and machine learning, conversational agents interpret questions and respond with messages using a simulated human tone (11). With increased online data availability and technological advances, commercially available VHAs have been marketed by companies such as Amazon (i.e., Echo/“Alexa”) and Google (i.e., Google Nest) for about 6 years. Commercially available voice-activated virtual home assistants (VHAs) are relatively inexpensive and may be particularly useful for older adults who have less technological literacy or vision or fine motor limitations.

Virtual home assistant users can listen to music, ask for information, and set reminders (12). Virtual home assistants also offer a range of applications or “skills” to engage users, such as games, that could serve as cognitive stimulation (13), mood enhancement (14), and relief from boredom (15). In addition to these common uses, VHAs offer a promising technology to provide social connectivity through video calling and surrogate companionship in a manner that addresses social isolation and helps relieve loneliness. The purpose of this mini review was to explore research findings on older adults' VHA use and its potential relationship to social isolation and loneliness.

## Materials and Methods

### Data Sources

A mini review was conducted to identify the current state of the science on older adults' VHA use and its potential relationship to social isolation and loneliness. Specific search terms were created for social isolation, loneliness, and older adults. However, as relatively new devices, no common electronic database terms for VHAs were identified. Thus, a variety of terms, including voice assistants, virtual assistants, and conversational agents were used in the searches. The search strategy was based on the recommended practice of each selected electronic database (PubMed, CINAHL, PsycInfo, Compendex) using a combination of sililar terms (Table 1).

**Table 1:** Concepts with MeSH and TIAB terms used for PubMed search.

**Construct/concept**	**Mesh term**	**TIAB term**
Voice-activated virtual home assistant	Ambient (sensors), artificial intelligence, deep learning, voice[–]activated virtual assistants, **conversational agents**, social support agents	Alexa, Google Home, Google Nest, Amazon Echo, digital assistant, virtual assistant, voice activated assistant, VHA
Social isolation	Isolation, social (use this word to find terms similar to “social isolation”), social exclusion, social alienation	COVID, social isolation, loneliness, social exclusion, social connectedness
Loneliness	Loneliness, depression, geriatric psychiatry	Social connectedness
Older adults	Older adults, gerontology, geriatrics	Grandparents, elderly, aging in place

### Data Selection and Extraction

Articles were deemed eligible for review if they were published in a peer-reviewed journal, written in English, addressed a concept related to social isolation or loneliness, and involved older adults' use of a commercially available VHA. Each eligible article was independently appraised based on inclusion and exclusion criteria by our team. Following title and abstract, the search yielded only four articles, and none were retained following full article review. Thus, the articles evaluated in this mini review were gleaned from the authors' reference libraries (*n* = 6) and article reference searches (*n* = 1).

## Results

### Study Characteristics

Study designs of the research included in this mini review were descriptive and used convenience samples. Except for one study (3), findings are based on qualitative content analysis. Of the seven studies, five used prospective methods and two were retrospective analyses of publicly available consumer reviews of VHAs (Table 2). The five prospective descriptive studies enrolled older adults who had not previously used VHAs and had small sample sizes. One study reported findings from 30 participants; however, not all participants were older adults (3). Another study included older adults (*n* = 10) and their respective support persons (*n* = 9) (12). All other sample sizes in the prospective studies included 7–12 older adult participants (aged 65–95 years). Study duration ranged from 3 weeks to 18 months. Amazon devices were used in four and Google devices in one of the prospective studies. The secondary analyses of consumer reviews reported user findings from Amazon Echo devices (13) and from Xiaomi XiaoAI, a VHA available in China (16).

**Table 2:** VHA study characteristics.

**Primary Author, Year**	**Study design**	**Sample**	**Participant ages**	**Study duration**	**Device**
Chambers, 2020	Prospective	*n* = 30 adults with chronic health conditions	Not reported	≥2 months	Echo Show
Chung, 2021	Retrospective	*n* = 320 consumer reviews from verified buyers, 2018	Not reported	N/A	Xiaomi XiaoAI
Corbett, 2021	Prospective	*n* = 19	Older adults (*n* = 10) 70 and older (X = 75); support persons (*n* = 9), (X = 53);	4 months	Echo Show and Dot (older adults), Echo Spot (support persons)
Kim, 2021	Prospective	*n* = 12	77–95 years	4 months	Google Home
O'Brien, 2020	Retrospective	*n* = 125 consumer reviews, 2015–2018	Not reported	N/A	Amazon Echo
Pradhan, 2019	Prospective	*n* = 7	65–83 years	3 weeks	Echo Dot
Scherr, 2020	Prospective	*n* = 11	68–83 years	≥12 months	Echo Show

*Chambers and Beaney (15); Chung and Woo (16); Corbett et al. (12); Kim and Choudhury (17); O'Brien et al. (13); Pradhan et al. (18); Scherr et al. (3)*.

### Social Isolation and Loneliness

Companionship was reported as a benefit for older adult VHA users in both consumer review studies. O'Brien et al.'s analysis of VHA consumer reviews was specific to older adults and companionship was one of five identified themes (13). Supporting quotes from the consumer reviews included: *Echo now keeps me company and allows me to keep my brain active too. She is more than a great bit of electronics….she is also a companion for me*. Chung and Woo's study analyzed comments from consumers of Xiaomi XiaoAI, were not specific to older adults (16). However, one theme noted by the investigators was the potential for VHA use to decrease loneliness and social isolation among older adult users, supported by the following consumer review: *Having her [Xiaomi XiaoAI], I am no longer lonely*.

The prospective studies (*n* = 5) identified companionship as a major finding. Chambers and Beaney's study provided VHAs to people who had health or dependence needs, of whom some were older adults (15). They reported that the participants who lived alone or were solitary for most of the day characterized the VHA as a source of companionship that reduced loneliness and improved mental health. Pradhan et al. conducted semi-structured interviews with older adult participants prior to installing the VHA devices in their homes and, after installation, conducted follow-up interviews every week for 3 weeks (18). One of their thematic findings was that the natural language processing and responsiveness of the VHA resulted in older adults perceiving the VHA as a friend. Supporting quotes included:

…*.when it talks, I don't see a box. I just see….It's like somebody is standing there talking to me….Somebody is here with me and they're having a conversation with me. It's making my day*. [(18), p. 2].

…*And it answers me and I am talking to it, I could think of it as a person*. [(18), p. 5].

Studies conducted by Corbett et al. (12) and Kim and Choudhury (17) placed VHAs in older adults' homes for 4 months. Kim and Choudhury interviewed participants every other week during the study whereas Corbett et al. interviewed participants once at the end of the 4-month study. Similar findings about companionship were obtained from each study as exemplified below.

*I have humanized that machine. I call her a ‘she' and a ‘her' and every morning I say*,

‘*Alexa what is the weather going to be like?' ….I always report in every morning and every night and I just have a kinship with Alexa.…. And you know, I know that's a machine…[laughs] but it's just that I feel like it's somebody here with me*. [(12), p. OA105].“*I think it is really good. It's not as if you're talking to yourself. You're talking to somebody. It makes you feel like you're really not alone. You never have to be alone because you can talk to Google”*. [(17), p. P8W8].

Authors of another study summarized the overall results from their study in a similar manner noting that Alexa had “*become a beloved new roommate*” for their participants. “Even though everyone knows that device is just a machine that can speak, for some of the participants it feels like an actual person that can reduce loneliness” [(3), p. 8].

Scherr et al. quantitatively measured loneliness and social isolation among their participants (*n* = 11) who were enrolled at least 12 months and lived within a defined neighborhood (3). Both individual and group interviews of participants were conducted. Group interviews allowed participants to interact with one another in person and exchange ideas about using the VHA. Every 3 months participants rated how often they felt lonely with response items that ranged from never to daily. Results indicated that participants reported reduced loneliness over time and reported increased social connectedness by using the VHA for video calling feature several times per week.

## Discussion

### Main Findings

The purpose of this mini review was to synthesize knowledge about the potential relationship between older adults' VHA use and the influence on social isolation and loneliness. The research findings were primarily based on qualitative content analysis. Quotes from several of the studies supported that the VHA reduced loneliness in many participants. Based on the findings of their study, Pradhan et al.'s suggested participants' interactions with the VHA reduced loneliness in the moment rather than alleviating a more global feeling of loneliness (18). However, Scherr et al. specifically measured both loneliness and social connectedness and reported participants improved in both areas over time (3).

All researchers in this mini review noted that the VHA provided a source of companionship for the users. “Companionship” is defined in the Meriam-Webster dictionary as “the good feeling that comes from being with someone else” (19) and “companion” is defined as “a person or animal you spend time with or enjoy being with…sometimes used figuratively” (20). The exemplar quotes reflected that the human-sounding voice and the conversational qualities of a Virtual home assistant lead users to personify the VHA and view it as a companion. Similar findings were documented in other VHA literature (21–24). Rubin et al. suggested that VHAs are inherently socially interactive because verbal prompting is required to activate the device (25). Other researchers reported that people in households of one or two people use VHAs more than those in larger households and attributed this finding to the social aspects of VHA use (26). Device placement in the home was also noted as important. The VHA may provide a human-like “presence” when the user is in the same room, but the feeling is reduced when the user is separated from the device (18).

To better understand VHA users' behavior, Han and Yang tested whether users may develop parasocial relationships with their VHAs (27). Parasocial relationship theory was originally used to explain people's imaginary interpersonal relationships between themselves and media (e.g., radio, television) personalities. Han and Yang measured the three parasocial relationship concepts of task attraction (how easy or worthwhile the device is to use and its reliability to complete a task), physical attraction (the user's perception of the visual appearance of the VHA), and social attraction (the user's intention to communicate and make friends with the VHA) in a sample of younger VHA users (*n* = 304). Users' social attraction to the VHA had four-fold greater impact on developing a positive parasocial relationship with the VHA than task attraction or physical attraction (27). Thus, their findings reinforce the importance of the human-like qualities of VHAs and provide insight into how people may develop the perception of the VHA as a companion, which may reduce loneliness.

Virtual home assistant capabilities that may reduce social isolation include providing information on news and current events, streaming religious services, and allowing voice and video calls to socially connect. Scherr et al. reported that many of their participants used the VHA at least several times per week for video calls (3). However, studies involving video calls to residents in long- term care facilities were inconclusive about the effect on social connectedness (28). Results of several studies in this mini review reported that participants liked the ease of using VHAs as compared to other technology, such as a mobile phone or computer (3, 12, 17), but also noted that older adult users desired more education and training about how to use it (12, 17). Thus, providing more training on VHA features than was provided in most of the studies in this mini review may help older adults to more fully realize the potential of the devices to reduce social isolation and loneliness (12, 17, 29, 30).

### Methodological Findings

All studies included in this mini review were exploratory or descriptive with small ( ≤30) sample sizes. Hence, the findings must be interpreted cautiously due to the voluntary, self-selected nature of the studies, and the lack of control groups. Further, only seven studies met the inclusion criteria, most of which were from the authors' libraries. One explanation for the lack of results from the database search is that VHAs have only been on the market for about 7 years so research on this topic is relatively scant. In addition, the devices are referred to by a plethora of other names in the literature, including digital assistants, conversational agents, and smart speakers. Consequently, database indexes do not have a consistent keyword for the devices, which limits the utility of systematic searches. Findings across studies consistently noted that VHA use offered companionship to older adults and may reduce social isolation and loneliness. Nonetheless, the small number and methodological characteristics of the studies reviewed portray that the state of the science of older adults' VHA use and its influence on social isolation and loneliness is in its infancy. Thus, there are many limitations to current knowledge, and the findings from the studies included in this mini review may not have adequately represented some of the risks and ethical controversies relevant to VHA use.

### Personal and Ethical Considerations

The findings of this mini review noted potential benefits of VHAs, but there are also potential risks associated with VHAs. Privacy concerns, often noted as a barrier to VHA use (24, 26, 31), did not emerge as a theme in the studies included in this mini review, possibly attributed to self-selection bias. Older adults who had privacy concerns (e.g., VHA is “always listening”) probably declined to participate in the studies, whereas those who participated had minimal concerns about privacy. Technological advances and regulatory safeguards are needed to mitigate privacy threats from VHA use (11, 24). The AI incorporated into VHAs is designed to mimic cognitive, emotional, and social intelligence, which contributes to the personification of VHAs (32) and to users viewing them as companions. Little is known about the eventual behavioral consequences of personifying a device (32), particularly when a person has cognitive impairment. Relatedly, an inability to remember the necessary commands to interact with a VHA may create frustration and agitation in people, particularly those with cognitive deficits (33). Conversely, AI is also being used to automate discourse analysis which may improve communication between people with dementia and their caregivers in the future (34). Concerns also exist about inequality and cultural and population biases built into technology-driven AI (35). For example, one study noted racial disparities in the automated speech recognition of VHAs (36). Alternatively, another study involving adults with intellectual disabilities had improvements in speech intelligibility after VHA interactions (37). Additionally, commercially available VHA devices are relatively affordable, but that benefit involves the risk of corporate-infused biases and targeted, personalized marketing opportunities (32), which may pose vulnerabilities for older adults. Virtual Home Assistant set-up and many functions require a smartphone and home internet access, which involves monthly costs. Taken together, the cost is prohibitive for some older adults (29). Thus, while the findings of the mini-review illustrate the potential benefits of VHAs for reducing social isolation and loneliness, there are also associated ethical considerations inherent to VHA use.

## Conclusions and Future Directions

Loneliness and social isolation are prevalent among older adults and pose serious health risks (5–7, 38). The results of this mini review suggest VHAs may offer a strategy to improve social connectedness and reduce loneliness for some older adults. A consistent finding was that many older adults perceived VHAs to be a companion. However, the state of the science is in its infancy. More research is needed to confirm these findings in larger, rigorously designed studies. Research is needed that quantitatively measures social isolation and loneliness outcomes among older adults using VHAs, as are studies that measure other known correlates to social isolation and loneliness, such as depressive symptoms (39), cognitive status (40), and functional ability (38). Additional research to define evidence-based strategies to teach VHA use skills to older adults is also needed. Ongoing refinements to the natural language processing features of VHAs to enhance the conversational experience will promote ease of use among older adults and improve the social attraction of the devices (17, 21), which may strengthen perceptions of the devices as a companion and reduce social isolation and loneliness. However, research to better understand the risks and benefits of using VHAs and other AI-infused technology is required. Public awareness of the potential risks and benefits is necessary for older adult users and other vulnerable populations to make informed choices (35). Continued research, public involvement in product development, and policy to promote ethical, unbiased AI that protects the privacy of users is necessary (35). In addition, the growth of all types of digital health necessitates devising strategies for affordable and reliable internet access to promote health equity (41, 42).

## Author Contributions

CC formed the concept of the mini review and identified the included studies in this mini review. CC and PW completed the database search, reviewed the literature, and drafted the mini review. All authors reviewed and edited the mini review.

## Funding

The University of South Carolina and the Advancing Chronic Care Outcomes through Research and Innovation Center provided partial support for this project.

## Conflict of Interest

The authors declare that the research was conducted in the absence of any commercial or financial relationships that could be construed as a potential conflict of interest.

## Publisher's Note

All claims expressed in this article are solely those of the authors and do not necessarily represent those of their affiliated organizations, or those of the publisher, the editors and the reviewers. Any product that may be evaluated in this article, or claim that may be made by its manufacturer, is not guaranteed or endorsed by the publisher.

## References

[1] Cudjoe TKM Roth DL Szanton SL. The epidemiology of social isolation: national health and aging trends study. J Gerontol B Psychol Sci Soc Sci. (2020) 75:107–13. 10.1093/geronb/gby037 29590462 PMC7179802

[2] Perissinotto CM Stijacic Cenzer I Covinsky KE. Loneliness in older persons a predictor of functional decline and death. Arch Intern Med. (2012) 172:1078–84. 10.1001/archinternmed.2012.1993 22710744 PMC4383762

[3] Scherr SA Meier A Cihan S. Alexa tell me more – about new best friends, the advantage of hands-free operation and life-long learning. In: Mensch und Computer 2020. Magdeburg (2020). 10.18420/muc2020-ws120-342

[4] Kotwal AA Holt-Lunstad J Newmark RL Cenzer I Smith AK Covinsky KE. Social isolation and loneliness among San Francisco Bay area older adults during the COVID-19 shelter-in-place orders. J Am Geriatr Soc. (2021) 69:20–9. 10.1111/jgs.16865 32965024 PMC7536935

[5] Holt-Lunstad J. The potential public health relevance of social isolation and loneliness: prevalence, epidemiology, and risk factors. Public Pol Aging Rep. (2017) 27:127–30. 10.1093/ppar/prx030

[6] Kuiper JS Zuidersma M Oude Voshaar RC Zuidema SU van den Heuvel ER Stolk RP. Social relationships and risk of dementia: a systematic review and meta-analysis of longitudinal cohort studies. Ageing Res Rev. (2015) 22:39–57. 10.1016/j.arr.2015.04.006 25956016

[7] Valtorta NK Kanaan M Gilbody S Ronzi S Hanratty B. Loneliness and social isolation as risk factors for coronary heart disease and stroke: systematic review and meta-analysis of longitudinal observational studies. Heart. (2016) 102:1009–16. 10.1136/heartjnl-2015-308790 27091846 PMC4941172

[8] Donovan NJ Blazer D. Social isolation and loneliness in older adults: review and commentary of a national academies report. Am J Geriatr Psychiatry. (2020) 28:1233–44. 10.1016/j.jagp.2020.08.005 32919873 PMC7437541

[9] Newall NEG Menec VH. Loneliness and social isolation of older adults: why it is important to examine these social aspects together. J Soc Pers Relat. (2017) 36:925–39. 10.1177/0265407517749045

[10] Schachner T Keller R von Wangenheim F. Artificial intelligence-based conversational agents for chronic conditions: systematic literature review. J Med Internet Res. (2020) 22:e20701. 10.2196/20701 32924957 PMC7522733

[11] McGreevey JD Hanson CW Koppel R. Clinical, legal, and ethical aspects of artificial intelligence-assisted conversational agents in health care. JAMA. (2020) 324:552–3. 10.1001/jama.2020.2724 32706386

[12] Corbett CF Combs EM Wright PJ Owens OL Stringfellow I Nguyen TP. Virtual home assistant use and perceptions of usefulness by older adults and support person dyads. Intl J Environ Res Public Health. (2021) 18:1113. 10.3390/ijerph18031113 33513798 PMC7908177

[13] O'Brien K Liggett A Ramirez-Zohfeld V Sunkara P Lindquist LA. Voice-controlled intelligent personal assistants to support aging in place. J Am Geriatr Soc. (2020) 68:176–9. 10.1111/jgs.16217 31617581

[14] Smith BJ Lim MH. How the COVID-19 Pandemic is focusing attention on loneliness and social isolation. Public Health Res Pract. (2020) 30:3022008. 10.17061/phrp3022008 32601651

[15] Chambers R Beaney P. The potential of placing a digital assistant in patients' homes. Br J Gen Pract. (2019) 70:8–9. 10.3399/bjgp20X707273 31879289 PMC6919512

[16] Chung S Woo BK. Using consumer perceptions of a voice-activated speaker device as an educational tool. JMIR Med Educ. (2020) 6:e17336. 10.2196/17336 32329740 PMC7210490

[17] Kim S Choudhury A. Exploring older adults' perception and use of smart speaker-based voice assistants: a longitudinal study. Comput Hum Behav. (2021) 124:106914. 10.1016/j.chb.2021.106914

[18] Pradhan A Findlater L Lazar A. “Phantom friend” or “just a box with information”: personification and ontological categorization of smart speaker-based voice assistants by older adults. Proc ACM Hum Comput Interact. (2019) 3:1–21. 10.1145/335931634322658

[19] Merriam-Webster's Learner's Dictionary. Companionship. (2021). Available online at: https://learnersdictionary.com/definition/companionship (accessed July 12, 2021).

[20] Merriam-Webster's Learner's Dictionary. Companion. (2021). Available online at: https://learnersdictionary.com/definition/companion (accessed July 12, 2021).

[21] Pradhan A Mehta K Findlater L. “Accessibility came by accident”: use of voice-controlled intelligent personal assistants by people with disabilities. In: 2018 CHI Conference on Human Factors in Computing Systems. Montreal, QC (2018). 10.1145/3173574.3174033

[22] Pradhan A Lazar A Findlater L. Use of intelligent voice assistants by older adults with low technology use. ACM Trans Comput Hum Interact. (2020) 27:1–27. 10.1145/3373759

[23] Purington A Taft JG Sannon S Bazarova NN Taylor SH. “Alexa is my new bff”: social roles, user satisfaction, and personification of the Amazon Echo. In: 2018 CHI Conference on Human Factors in Computing Systems. Denver, CO (2017). 10.1145/3027063.3053246

[24] Paay J Kjeldskov J Hansen KM Jørgensen T Overgaard KL. Digital ethnography of home use of digital personal assistants. Behav Inf Technol. (2020) 2020, 1–19. 10.1080/0144929X.2020.1834620

[25] Rubin V Chen Y Thorimbert LM. Artificially intelligent conversational agents in libraries. Library Hi Tech. (2010) 28:496–522. 10.1108/07378831011096196

[26] McLean G Osei-Frimpong K. Hey Alexa…examine the variables influencing the use of artificial intelligent in-home voice assistants. Compu Hum Behav. (2019) 99:28–37. 10.1016/j.chb.2019.05.009

[27] Han S Yang H. Understanding adoption of intelligent personal assistants: a parasocial relationship perspective. Ind Manag Data Syst. (2018) 118:618–36. 10.1108/IMDS-05-2017-0214

[28] Noone C McSharry J Smalle M Burns A Dwan K Devane D. Video calls for reducing social isolation and loneliness in older people: a rapid review. Cochrane Database Syst Rev. (2020) 5:CD013632. 10.1002/14651858.CD013632 32441330 PMC7387868

[29] Knowles B Hanson VL. The wisdom of older technology (non)users. Commun ACM. (2018) 61:72–7. 10.1145/3179995

[30] Kowalski J Jaskulska A Skorupska K Biele KAC Kopec W Marasek K. Older adults and voice interaction: a pilot study with google home. In: CHI EA '19: Extended Abstracts of the 2019 CHI Conference on Human Factors in Computing Systems. Glasgow (2019). 10.1145/3290607.3312973

[31] Kim S. Exploring how older adults use a smart speaker-based voice assistant in their first interactions: qualitative study. JMIR mHealth uHealth. (2021) 9:e20427. 10.2196/20427 33439130 PMC7840274

[32] Ameen N Hosany S Tarhini A. Consumer interaction with cutting-edge technologies: implications for future research. Comput Human Behav. (2021) 120:106761. 10.1061/j.chb.2021.106761

[33] Ruggiano N Brown EL Roberts L Suarez CVF Luo Y Hao Z. Chatbots to support people with dementia and their caregivers: systematic review of functions and quality. JMIR. (2021) 23:e25006. 10.2196/25006 34081019 PMC8212632

[34] Whelan B-M Angus D Wiles J Chenery HJ Conway ER Copland DA. Toward the development of SMART communication technology: automating the analysis of communicative trouble and repair in dementia. Innovation in Aging. (2018) 3:igy034. 10.1093/geroni/igy034 30539162 PMC6276976

[35] Zidaru T Morrow EM Stockley R. Ensuring patient and public involvement in the transition to AI-assisted mental health care: a systematic scoping review and agenda for design justice. Health Expect. (2021) 24:1072–124. 10.1111/hex.13299 34118185 PMC8369091

[36] Koenecke A Nam A Lake E Nudell J Quartey M Mengesha Z. Racial disparities in automated speech recognition. Proc Natl Acad Sci USA. (2020) 117:7684–9. 10.1073/pnas.1915768117 32205437 PMC7149386

[37] Smith E Sumner P Hedge C Powell G. Smart speaker devices can improve speech intelligibility in adults with intellectual disability. Int J Lang Commun Disord. (2021) 56:583–93. 10.1111/1460-6984.12615 33772998

[38] O'Suilleabhain PS Gallagher S Steptoe A. Loneliness, living alone, and all-cause mortality: the role of emotional and social loneliness in the elderly during 19 years of follow-up. Psychosom Med. (2021) 81:5212–526. 10.1097/PSY.0000000000000710 31094903 PMC6615929

[39] Ge L Yap CW Ong R Heng BH. Social isolation, loneliness and their relationships with depressive symptoms: a population-based study. PLoS ONE. (2017) 12:e0182145. 10.1371/journal.pone.0182145 28832594 PMC5568112

[40] Yang R Wang H Edelman LS Tracy EL Demiris G Sard KA. Loneliness as a mediator of the impact of social isolation on cognitive functioning of Chinese older adults. Age Ageing. (2020) 49:599–604. 10.1093/ageing/afaa020 32147683

[41] Davies AR Honeyman M Gann B. Addressing the digital inverse care law in the time of COVID-19: potential for digital technology to exacerbate or mitigate health inequalities. J Med Internet Res. (2021) 23:e21726. 10.2196/21726 33735096 PMC8030655

[42] Tappen RM Cooley ME Luckmann R. Panday S. Digital health information disparities in older adults: a mixed methods study. J Racial Ethn Health Disparities. (2021) 2021:1–11. 10.1007/s40615-020-00931-3 33415705 PMC7790471

